# A peptide mimicking the binding sites of VEGF-A and VEGF-B inhibits VEGFR-1/-2 driven angiogenesis, tumor growth and metastasis

**DOI:** 10.1038/s41598-018-36394-0

**Published:** 2018-12-18

**Authors:** Maryam Farzaneh Behelgardi, Saber Zahri, Farhad Mashayekhi, Kamran Mansouri, S. Mohsen Asghari

**Affiliations:** 1 Department of Biology, Faculty of Science, University of Mohaghegh Ardabili, Ardabil, Iran; 2 Department of Biology, Faculty of Sciences, University of Guilan, Rasht, Iran; 3 Medical Biology Research Center, Kermanshah University of Medical Sciences, Kermanshah, Iran

## Abstract

Interfering with interactions of vascular endothelial growth factors (VEGFs) with their receptors (VEGFRs) effectively inhibits angiogenesis and tumor growth. We designed an antagonist peptide of VEGF-A and VEGF-B reproducing two discontinuous receptor binding regions of VEGF-B (loop 1 and loop3) covalently linked together by a receptor binding region of VEGF-A (loop3). The designed peptide (referred to as VGB4) was able to bind to both VEGFR1 and VEGFR2 on the Human Umbilical Vein Endothelial Cells (HUVECs) surface and inhibited VEGF-A driven proliferation, migration and tube formation in HUVECs through suppression of ERK1/2 and AKT phosphorylation. The whole-animal fluorescence imaging demonstrated that fluorescein isothiocyanate (FITC)-VGB4 accumulated in the mammary carcinoma tumors (MCTs). Administration of VGB4 led to the regression of 4T1 murine MCT growth through decreased expression of p-VEGFR1 and p-VEGFR2 and abrogation of ERK1/2 and AKT activation followed by considerable decrease of tumor cell proliferation (Ki67 expression) and angiogenesis (CD31 and CD34 expression), induction of apoptosis (increased p53 expression, TUNEL staining and decreased Bcl2 expression), and suppression of metastasis  (increased E-cadherin and decreased N-cadherin, NF-κB and MMP-9 expression). These findings indicate that VGB4 may be applicable for antiangiogenic and antitumor therapy.

## Introduction

Given uncontrolled cell proliferation of tumor tissue, new vascular growth occurs at high levels for further providing oxygen and nutrient supply for the fast-growing tumor cells^1^. New blood vessels formation (angiogenesis) is controlled through the balance between pro- and anti-angiogenic factors so that the breakage of this balance leads to tumor growth^1^. Vascular endothelial growth factor (VEGF), a pro-angiogenic factor secreted by endothelial and tumoral cells, has the prominent role in tumor angiogenesis, growth and metastasis^2,3^.

The VEGF family exerts their biological functions through the interaction with transmembrane receptors such as tyrosine kinase receptors VEGFR1 and VEGFR2. The ligands which specifically bind to VEGFR1 are VEGF-A, -B and PlGF while those bind to VEGFR2 are VEGF-A, -C, -D and –E^4,5^. Binding of VEGFs to VEGF receptor-1 and -2 triggers downstream signaling pathways resulted in EC proliferation, migration, invasion and high vascular permeability through the signaling molecules such as ERK1/2 and AKT^6–9^. Thus, therapeutic angiogenesis studies have been mostly focused on the disruption of VEGF-VEGFR pathways.

The shortcomings of anti-VEGF or anti-VEGF receptor antibodies and tyrosine kinase inhibitors, having some drawbacks including inappropriate pharmacokinetics (Abs) and low specificity (TKIs), have limited their clinical outcomes^10,11^. On the other hand, peptides, as a new class of therapeutics, has been considered as an intense research subject. Many peptides with superior pharmacokinetic properties have been developed to block protein-protein interactions^11^. Rationally designed peptides can mimic the binding regions in complex protein-protein and antagonize a biological activity of target protein with high specificity^12^. In recent years, many researchers have designed a number of VEGF or VEGFRs antagonist peptides through a rational approach^13–19^. In the present study, given that signaling through both VEGFR1 and VEGFR2 is crucial for tumor angiogenesis, growth and metastasis^20^, a linear peptide was rationally designed from α2-β3 loop (loop1) and β5-β6 loop (loop3) of VEGF-B as well as β5-β6 loop (loop3) of VEGF-A, according to their complex with VEGFR1 D2 and VEGFR2 D2. The designed peptide, denoted as VGB4, recognized both VEGFR1 and R2. Based on *in vitro* and *in vivo* studies, VGB4 potently inhibited proliferation, migration and tube formation of human umbilical vein endothelial cells (HUVECs), as well as 4T1 mammary carcinoma tumor (MCT) angiogenesis, growth and metastasis. These results suggest that VGB4 is a potential candidate for future clinical investigations.

## Results

### Peptide design

**Figure 1:**
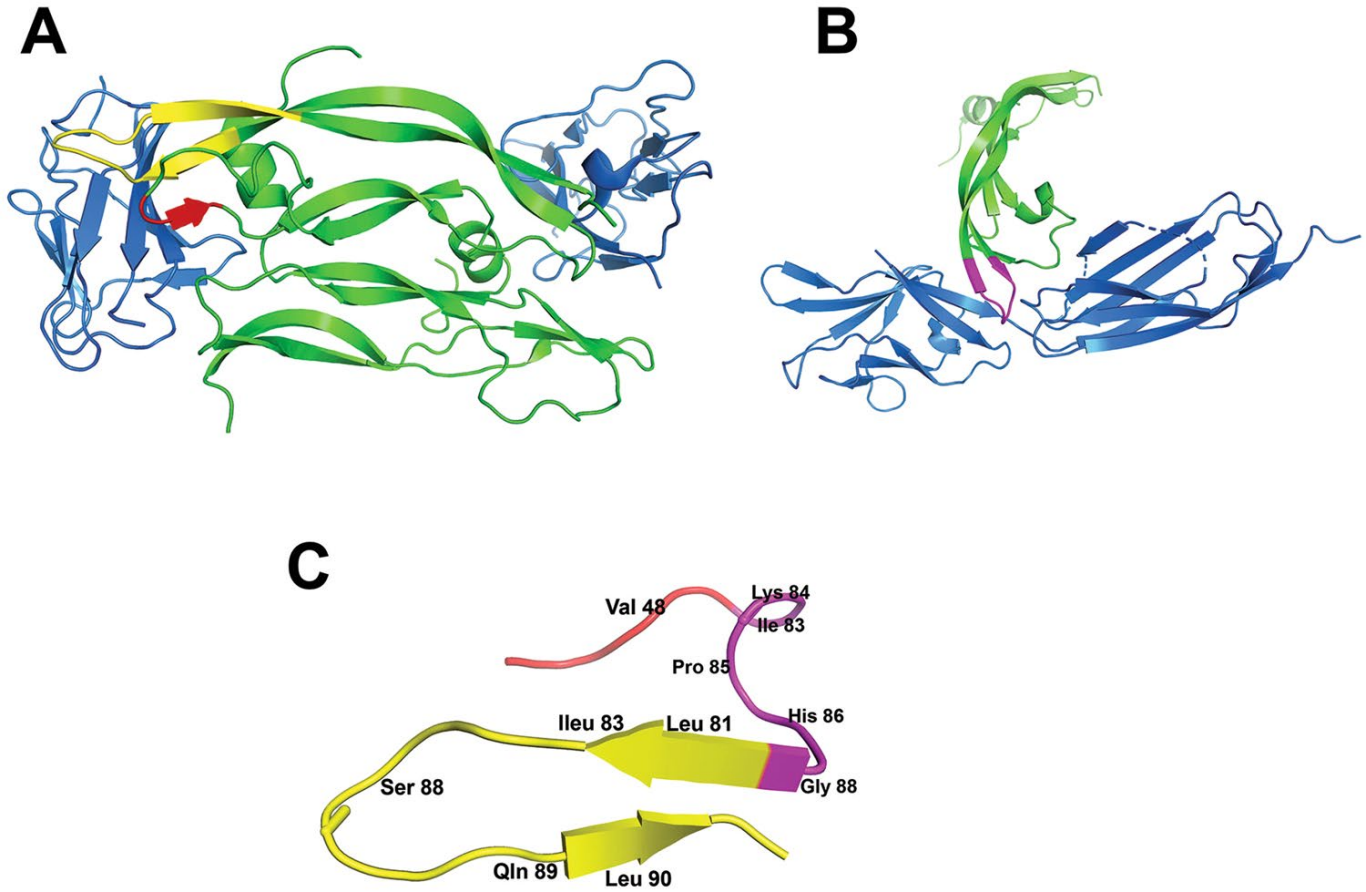
Ribbon representation of VEGFB-VEGFR1 D2 complex (**A**), VEGFA-VEGFR2 D2 complex (**B**) and schematic representation of the structure of VGB4 peptide (**C**). Individual segments are highlighted in different colors: VEGFR1 D2 and VEGFR2 D2 are represented in blue whereas VEGF monomers are colored in green. The interacting regions in VEGFB-VEGFR1 D2 complex are colored in red and yellow. The interacting region in VEGFA-VEGFR2 D2 complex is colored in violet. The Figure was made using the software PyMOL (PYMOL Molecular Graphics System, Version 2.1 Schrödinger, LLC).

The blockage of either VEGFR1 or VEGFR2 has been shown to effectively inhibit tumor angiogenesis^21–23^. However, their downstream signaling pathways have convergence and cross-activation, resulting in development of resistance to therapeutics targeting only one receptor tyrosine kinase^24^. Therefore, dual blockade of VEGFR1 and VEGFR2 is required for acquisition of better efficacy^20^. The aim of the present study was to rationally design a peptide that simultaneously binds and blocks both VEGFR1 and VEGFR2. The crystal structure of the complex between VEGF-B and the extracellular domain of VEGFR1 revealed that the β-hairpin fragment 79–93 and the segment 45–48 within α2-β3 loop in VEGF-B are in close proximity, forming an important binding interface with the second domain of VEGFR1 (VEGFR1 D2)^25^. Coincidentally, segment 79–93 belonging to VEGF-A, especially residues 83–88, is involved in the interaction with VEGFR2 D2 and D3^26–28^. Accordingly, VEGF-B segments 45–48 and 79–93, including binding residues Val48, Leu81, Ile83, Ser88, Gln89 and Leu90, and VEGF-A segment 83–88, comprising binding residues Ile83, Lys84, Pro85, His86 and Gly88, were selected to be covalently linked into a single peptide. The VEGF-A segment (turn) flanked by two VEGF-B segments at N- and C-terminal sides (loop and β-hairpin, respectively). Finally, Gly91 was removed from VEGF-B segment because of low intrinsic propensity to form β-sheet structure, and Gln87 was removed from VEGF-A segment as this residue does not participate in the interaction with VEGFR2 (Fig. 1). The sequence of the designed 23-amino acid peptide (referred to as VGB4) was _2_HN-KQLVIKPHGQILMIRYPSSQLEM-COOH. Corresponding scrambled control peptide (referred to as scr), containing the same amino acids as peptide VGB4 in a random order (_2_HN-KPIYSKPRIQMHMQILEQVKSGL-COOH), was also synthesized and characterized.

### VGB4 binding to VEGFR1 and VEGFR2

**Figure 2:**
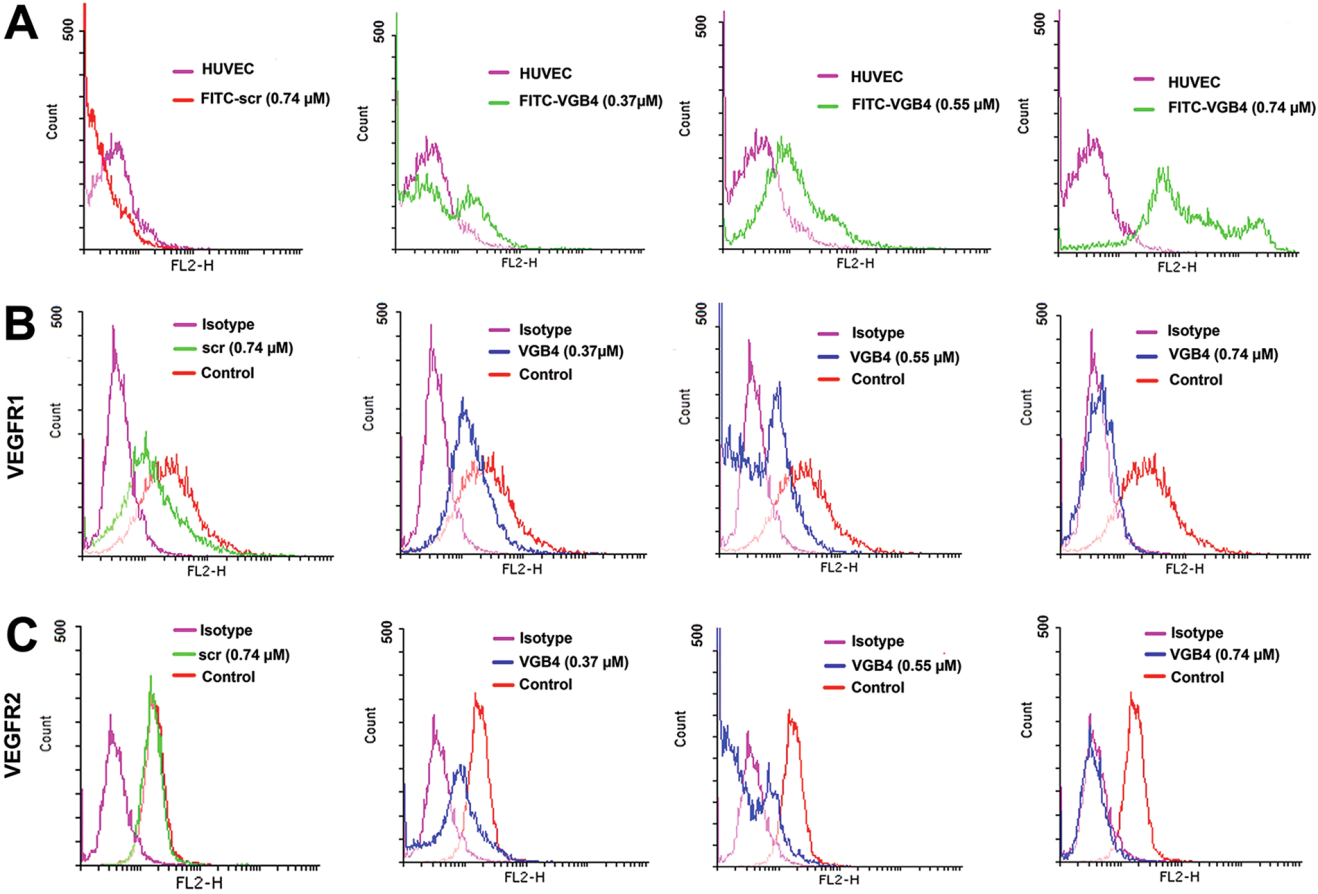
Receptor binding assays using flow cytometry. (**A**) HUVECs were incubated with different concentrations of FITC-VGB4 peptide (0.37, 0.55 and 0.74 μM) (green curve) or FITC-scr peptide (0.74 μM) (red curve). Autofluorescence of the cells are reported in violet. (**B**) and (**C**) HUVECs were incubated with different concentrations of VGB4 peptide (0.37, 0.55 and 0.74 μM) (blue curve) or scr peptide (0.74 μM) (green curve) for over night in the dark. After adding anti-VEGFR1 and anti-VEGFR2 antibodies, flow cytometric analysis was performed using a BD FACSCalibur Flow Cytometer (see Materials and Methods for details).

To assess the cell-surface binding capability of VGB4, HUVECs were incubated with different concentrations of fluorescein isothiocyanate (FITC)-conjugated VGB4 (0.37, 0.55 and 0.74 μM) or FITC-conjugated scr peptide (0.74 μM) and analyzed by flow cytometry. As shown in Fig. 2A, with increasing the concentration of FITC-VGB4, intensity of fluorescence was markedly increased compared to untreated HUVECs and scr peptide. To confirm whether VGB4 binding was attributed to VEGF receptors, HUVECs were incubated with anti-VEGFR1 primary antibody and Phycoerythrin (PE)-labeled goat anti-mouse IgG secondary antibody or anti-VEGFR2 primary antibody and FITC-labeled rabbit anti-mouse IgG secondary antibody and increasing concentrations of VGB4 (0.37, 0.55 and 0.74 μM) or scr (0.74 μM). As shown in Fig. 2B,C, incubation with VGB4 led to a considerable reduction of fluorescent signals in a dose-dependent manner when compared to control and scr peptide (0.74 μM). These results show that VGB4 can bind to both VEGFR1 and VEGFR2 on the surface of endothelial cells and compete with antibodies that recognize the ectodomains of the receptors.

**Figure 3:**
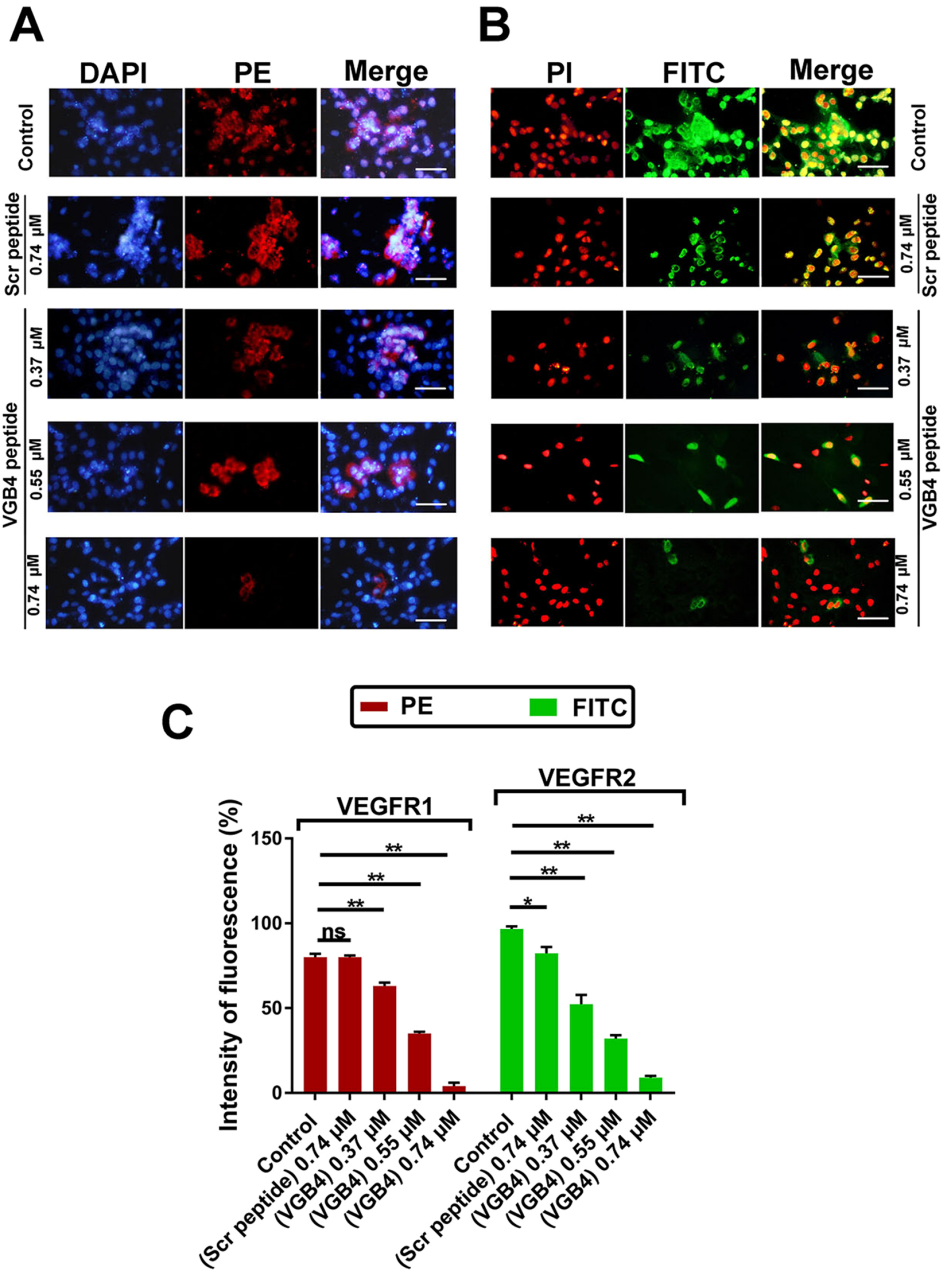
The binding properties of VGB4 to VEGFR1 and VEGFR2  using immunofluorescence. HUVECs were treated for overnight with VGB4, fixed and stained with (**A**) anti-VEGFR1 –primary antibody which was detected with PE-labeled goat anti-mouse IgG secondary antibody, and (**B**) anti-VEGFR2 primary antibody which was detected with FITC-labeled rabbit anti-mouse IgG secondary antibody. (**C**) Graph representing the percent of fluorescence intensity in treated groups compared to control and scr peptide. All data were represented as mean ± SD of three experiments. *P ≤ 0.05, **P ≤ 0.001 versus control. All images were taken by Olympus fluorescence microscope and magnification 400×; bar = 50 µm.

**Figure 4:**
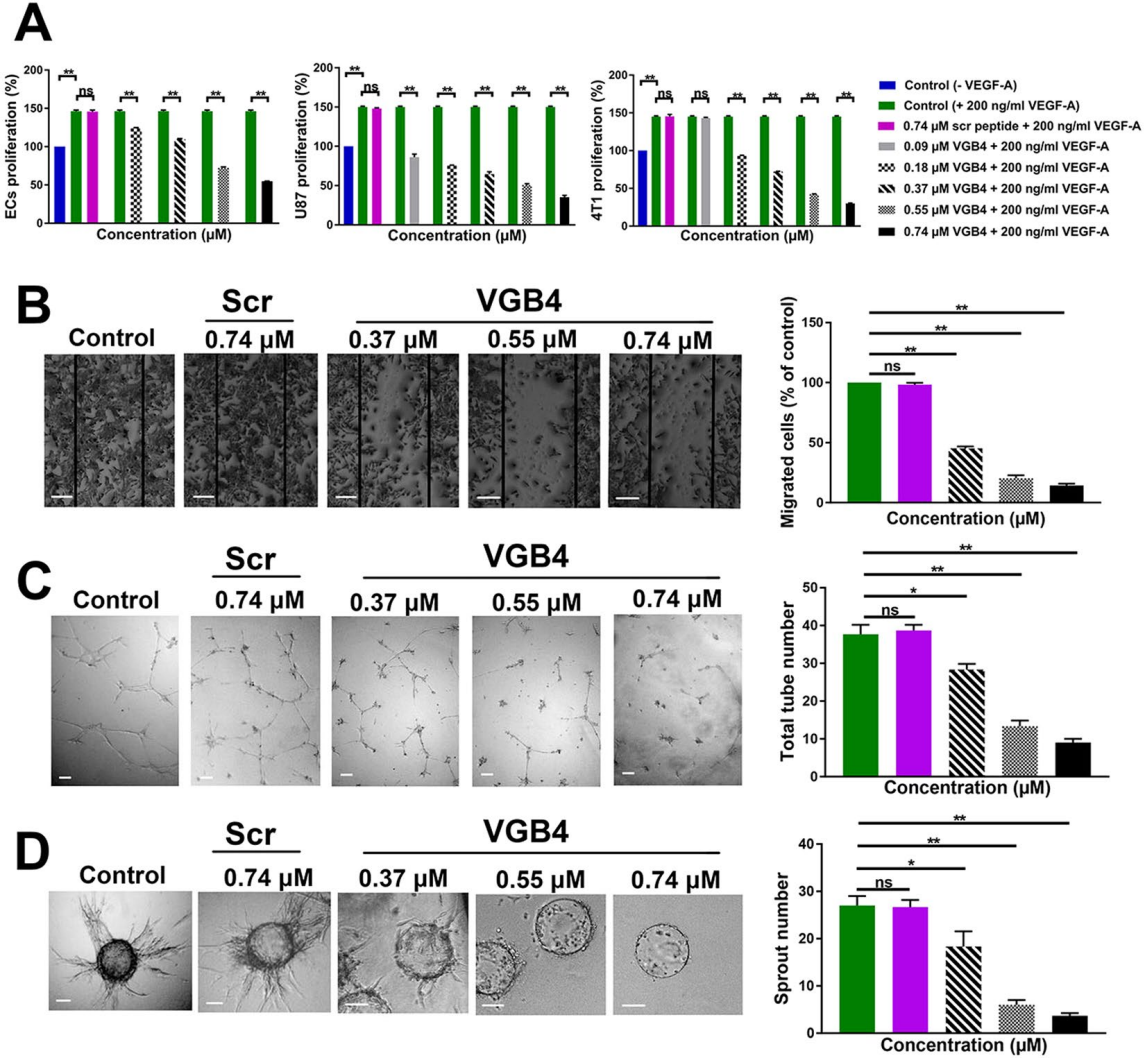
The effect of VGB4 on the cell proliferation, migration, tube formation and intracellular signaling *in vitro*. (**A**) HUVEC, 4T1 and U87 cells were treated with different concentrations of VGB4 for 36 h in the presence of 200 ng/ml VEGF-A. The obtained IC_50_ values of VGB4 by VEGF-A-induced HUVEC, 4T1 and U87 cell proliferation assays were at 0.55, 0.37 and 0.18 μM, respectively. (**B**) Representative images and quantitative data of scratch wound-healing migration in HUVECs. The monolayer of HUVECs were scratched using the sterile pipette tip and treated with different concentrations of VGB4 for 24 h following VEGF-A (200 ng/ml) stimulation. (**C**) Representative images and quantitative data of the tube-like structure formation assay of HUVECs in control and VGB4-treated groups following VEGF-A (200 ng/ml) stimulation. (**D**) Representative images and quantitative data of the spheroid angiogenesis (3D) assay acquired after coating HUVECs on to cytodex micro carrier beads and embedded in to the collagen gel. The sprout formation was evaluated for treated cells and control in the presence of different VGB4 concentrations and 200 ng/ml VEGF-A. (Magnification 10×; bar = 50 µm). All data were represented as mean ± SD, n = 3, *P ≤ 0.05, **P ≤ 0.001 compared to the control.

Then, the capability of VGB4 to bind to VEGFR1 and VEGFR2 on the surface of endothelial cells was verified using fluorescence microscopy.  For this purpose, HUVECs were incubated with the increasing concentrations of VGB4 (0.37, 0.55 and 0.74 μM) accompanied by adding the anti-VEGFR1 primary antibody and PE-labeled goat anti-mouse IgG secondary antibody. As shown in Fig. 3A, VGB4 reduced the fluorescence intensity in a dose-dependent manner. For instance, at 0.74 μM concentration of VGB4, the fluorescence intensity was reduced by 5% compared to 76% in the control (Fig. 3C). These results verify that VGB4 recognizes VEGFR1. To further confirm the VEGFR1 binding property of VGB4, its ability to inhibit the proliferation of 4T1 mammary carcinoma cell line, which highly expresses VEGFR1, was tested. Obviously, VGB4 inhibited VEGF-A induced proliferation of 4T1 cells with a half maximal inhibitory concentration (IC_50_) of ~0.37 μM (Fig. 4A), which verifies the ability of VGB4 to bind to VEGFR1. Then, VGB4 binding to VEGFR2 on HUVECs surface was evaluated by florescence microscopy. For this purpose, after adding the different concentrations of VGB4, anti-VEGFR2 primary antibody and FITC-labeled rabbit anti-mouse IgG secondary antibody, the fluorescence intensity of FITC was quantified. Interestingly, VGB4 demonstrated dose-dependent binding to VEGFR2 on HUVECs, with a significant reduction in fluorescence intensity (11%) at 0.74 μM of VGB4 compared to control (97%) (Fig. 3B,C). In addition, as shown in Fig. 4A, VGB4 inhibited VEGF-A induced proliferation of U87 glioblastoma cell line mostly expressing VEGFR2 on the cell surface, with an IC_50_ ~ 0.18 μM. These results confirm that VGB4 is able to bind to VEGFR2 as well.

### Inhibition of HUVECs proliferation, migration and tube formation by VGB4

Based on the results obtained from the binding studies, VGB4 recognizes both VEGFR1 and VEGFR2. To test whether blockade of VEGFR1 and VEGFR2 leads to the inhibition of angiogenesis, we evaluated the effect of VGB4 on different aspects of angiogenesis, including proliferation, migration and tube formation after stimulation with a high concentration of VEGF-A (200 ng/ml). Proliferation of HUVECs was increased by 46% in the presence compared to the absence of VEGF-A (*P ≤ *0.001) (Fig. 4A). VGB4 led to a dose-dependent suppression of VEGF-A induced EC proliferation and the inhibition was about 63% at 0.74 μM (*P ≤ *0.001). In addition, VGB4 showed the IC_50_ value of 0.55 μM in HUVECs. Scr peptide (0.74 μM) could not inhibit VEGF-A-induced EC proliferation. Then, the wound healing assay was used to evaluate the anti-migratory property of VGB4. In the presence of VEGF-A, untreated HUVECs almost completely migrated to the wound area after 24 h, but VGB4 treatment significantly decreased VEGF-A-induced migration by 79.7% and 85.7% at 0.55 μM and 0.74 μM, respectively, when compared to control and scr-treated cells (*P ≤ *0.001) (Fig. 4B). To further investigate the effect of VGB4 on EC behavior, we performed two and three dimensional tube formation assays. As shown in Fig. 4C, VGB4 could inhibit VEGF-A-induced two-dimensional tube formation in a dose-dependent manner. A statistically significant decreased number of capillary-like tubes by 64.7% and 76.2% was observed at 0.55 μM and 0.74 μM, respectively, as compared to control and scr (*P ≤ *0.001). In addition, VGB4 inhibited VEGF-A-induced sprouting angiogenesis in collagen matrix with the maximum reduction effect on sprout number by 67.5% and 84.1% at 0.55 μM and 0.74 μM, respectively, as compared to control and scr (*P ≤ *0.001) (Fig. 4D). These results reflect the anti-angiogenic property of VGB4 in HUVECs.

###  The tumor-accumulating ability of VGB4 investigated by *in vivo* imaging

**Figure 5:**
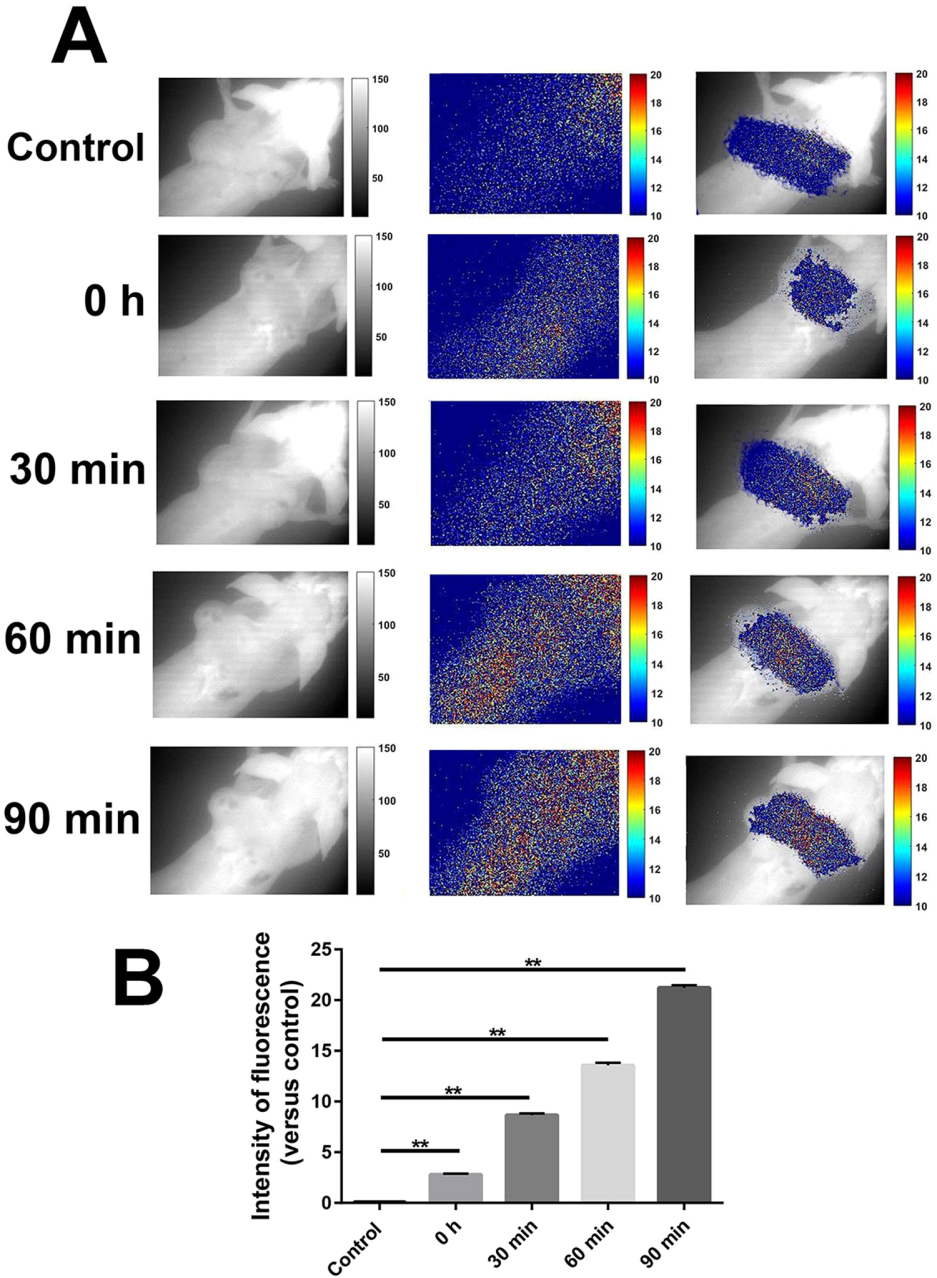
The investigation of the tumor-accumulating ability of VGB4 using 2D optical imaging. (**A**)  After intravenous VGB4-FITC injection into BALB/c mice bearing 4T1 murine MCT, the whole body of each mouse was scanned at 0, 30, 60 and 90 minutes after injection. (**B**) The fluorescence intensity (in tumor region) at different times (0, 30, 60 and 90 minutes) after injection was compared to control group (untreated animals). The experiment was repeated three times. **P ≤ 0.001, compared to the control group.

The accumulation of VGB4 peptide in 4T1 murine MCT was assessed using 2D optical imaging. A significant increase of fluorescence intensity was observed at 30, 60 and 90 minutes after VGB4-FITC injection compared to control (Fig. 5) (*P* ≤ 0.001). Importantly, no significant increase of fluorescence intensity in tumor region was observed even at 90 minutes after FITC-scr injection compared to control (data not shown).

### Regression of 4T1 murine MCT growth by VGB4

**Figure 6:**
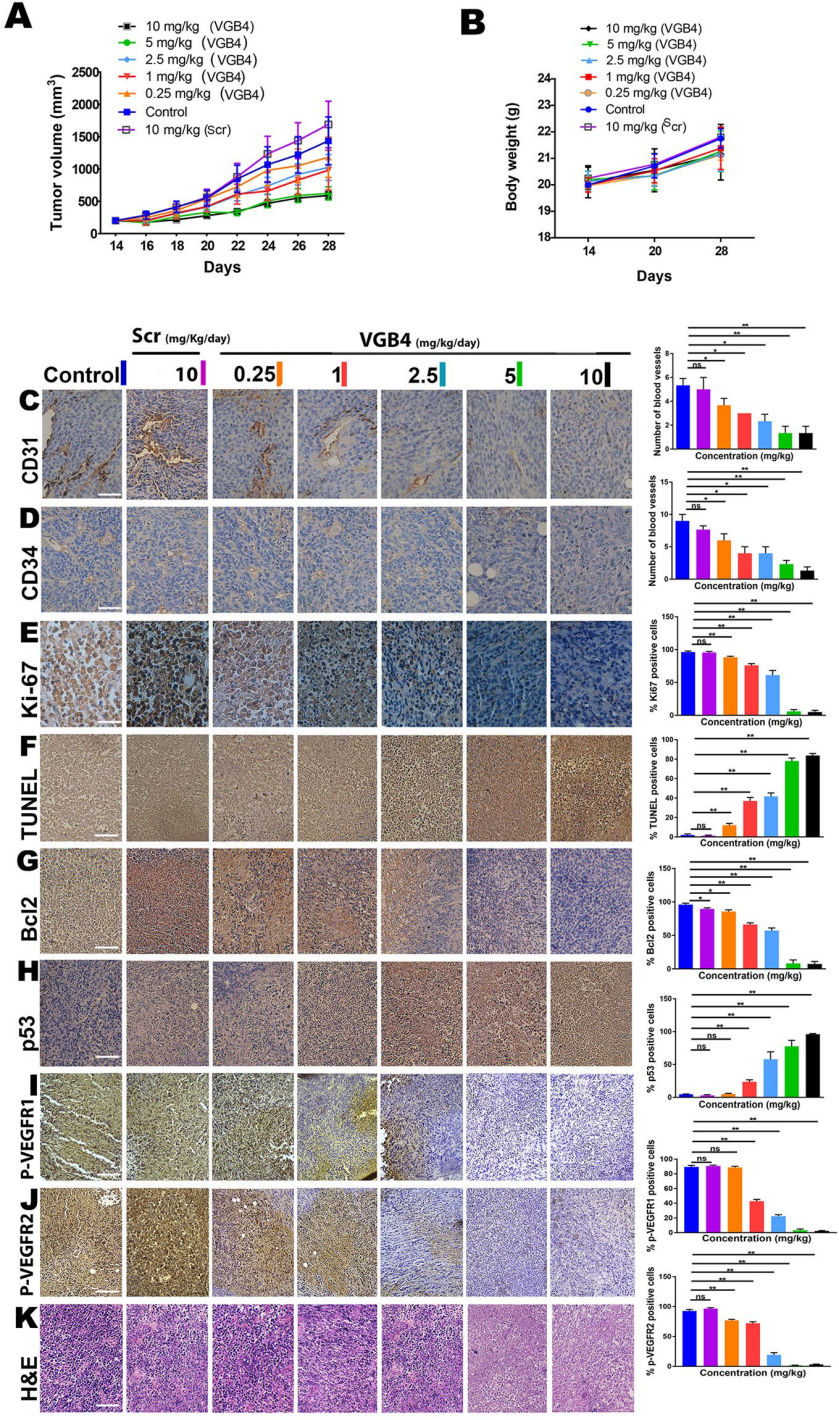
The inhibitory effect of VGB4 on tumor growth, metastasis and signaling pathways *in vivo*. (**A**) 4T1  murine MCT was transplanted into female BALB/c mice. On day 14 after transplantation (when the tumor volume reached ~200 mm^3^), animals in the experimental groups were treated daily with intraperitoneal (i.p.) injection of different doses (0.25, 1, 2.5, 5 and 10 mg/kg/day) of VGB4 or (10 mg/kg) scr, whereas control mice received equal volume of PBS (n = 7 mice for each group). (**B**) The average body weight of each group was measured on days 14, 20 and 28 then expressed as the mean ± SD (n = 7 per group). (**C**–**K**) Immunohistochemical analyses. Representative images and quantitative analysis of (**C**) CD31, (**D**) CD34, (**E**) Ki-67 staining (Magnification 40×; bar = 20 µm) and (**F**) TUNEL, (**G**) Bcl2, (**H**) P53, (**I**) p-VEGFR1, (**J**) p-VEGFR2 and (**K**) H&E staining (Magnification 10×; bar = 100 µm) of VGB4 treated tumors and PBS-treated tumors 28 days after subcutaneous transplantation of 4T1 MCTs in BALB/c mice. All data were represented as mean ± SD, n = 3, *P ≤ 0.05, **P ≤ 0.001, compared to the control group.

The antitumor property of VGB4 was examined by subcutaneous transplantation of 4T1  murine MCTs into BALB/c mice. Different doses of VGB4 (0.25, 1, 2.5, 5 and 10 mg/kg/day) or scr (10 mg/kg) were intraperitoneally (i.p.) administrated for two weeks, starting at day 14 after tumor transplantation. After 14 days of treatment, VGB4-treated BALB/c mice had a significant reduction of tumor growth of 18%, 29%, 32%, 57% and 59% with doses of 0.25, 1, 2.5, 5 and 10 mg/kg of VGB4 peptide, respectively (Fig. 6A). Statistically significant inhibition of tumor growth was observed at the doses of 5 mg/kg and 10 mg/kg on 28th day compared to the control group (*P* = 0.024). Given that increasing the peptide dosage from 5 to 10 mg/kg had no significant antitumor efficacy, the maximum effective dose of VGB4 seems to be at 5 mg/kg in this tumor model. In contrast to VGB4, treatment of BALB/c mice with 10 mg/kg of scr peptide could not inhibit tumor growth during two weeks of treatment. Importantly, no mortality was observed in the animals during treatment period (data not shown). Moreover, the body weight of mice was increased during 14 days of the peptide treatment (Fig. 6B). These results suggest that the peptide is nontoxic at the dosages used.

### Immunohistochemical analyses

To further disclose the antitumor mechanism of VGB4, the immunohistochemical markers CD31, CD34, Ki-67, Bcl2, P53, p-VEGFR1 and p-VEGFR2 were examined in tumor tissues in each group on the last day of the peptide administration (day 28). Analysis of microvessel density (MVD) by CD31 and CD34 staining revealed that, in contrary to scr peptide, all treated doses of VGB4 resulted in a significant reduction in tumor vessels as compared to PBS-treated controls and the increase of the peptide dosage led to more striking reduction of tumor vessels (Fig. 6C,D). Ki-67 (as a proliferative index) staining revealed significant changes in cell proliferation between the treated and control groups: the control group showed strikingly high Ki-67 expression, indicating growth and proliferation of tumor cells; a considerable decrease in Ki-67 expression was observed in all VGB4-treatedt groups but not in scr peptide treated group (*P* ≤ 0.05 for 0.25 mg/kg, and *P* ≤ 0.001 for 1, 2.5, 5 and 10 mg/kg) (Fig. 6E). Then, TUNEL, Bcl2 and P53 staining were performed for examination of apoptosis in tumor tissues. The proportion of TUNEL-positive cells in all treatment groups was significantly higher than that in control and scr peptide-treated groups (*P ≤ *0.001) (Fig. 6F). Likewise, VGB4 significantly decreased the expression of Bcl2 in all treatment groups compared to control and scr peptide treated groups (*P* ≤ 0.05 for 0.25 mg/kg, and *P* ≤ 0.001 for 1, 2.5, 5 and 10 mg/kg) (Fig. 6G). Furthermore, VGB4 markedly increased the expression level of P53 at doses of 1, 2.5, 5 and 10 mg/kg in treated tumors compared to control and scr peptide treated groups (*P* ≤ 0.001) (Fig. 6H). VGB4 strongly decreased the expression level of phospho-VEGFR1 (P-VEGFR1) and phospho-VEGFR2 (P-VEGFR2) (Fig. 6I,J). In agreement with these results, H&E staining revealed noticeable morphological changes in the treatment compared to the control groups (Fig. 6K).

### Effect of VGB4 on intracellular signaling pathways associated with VEGF

**Figure 7:**
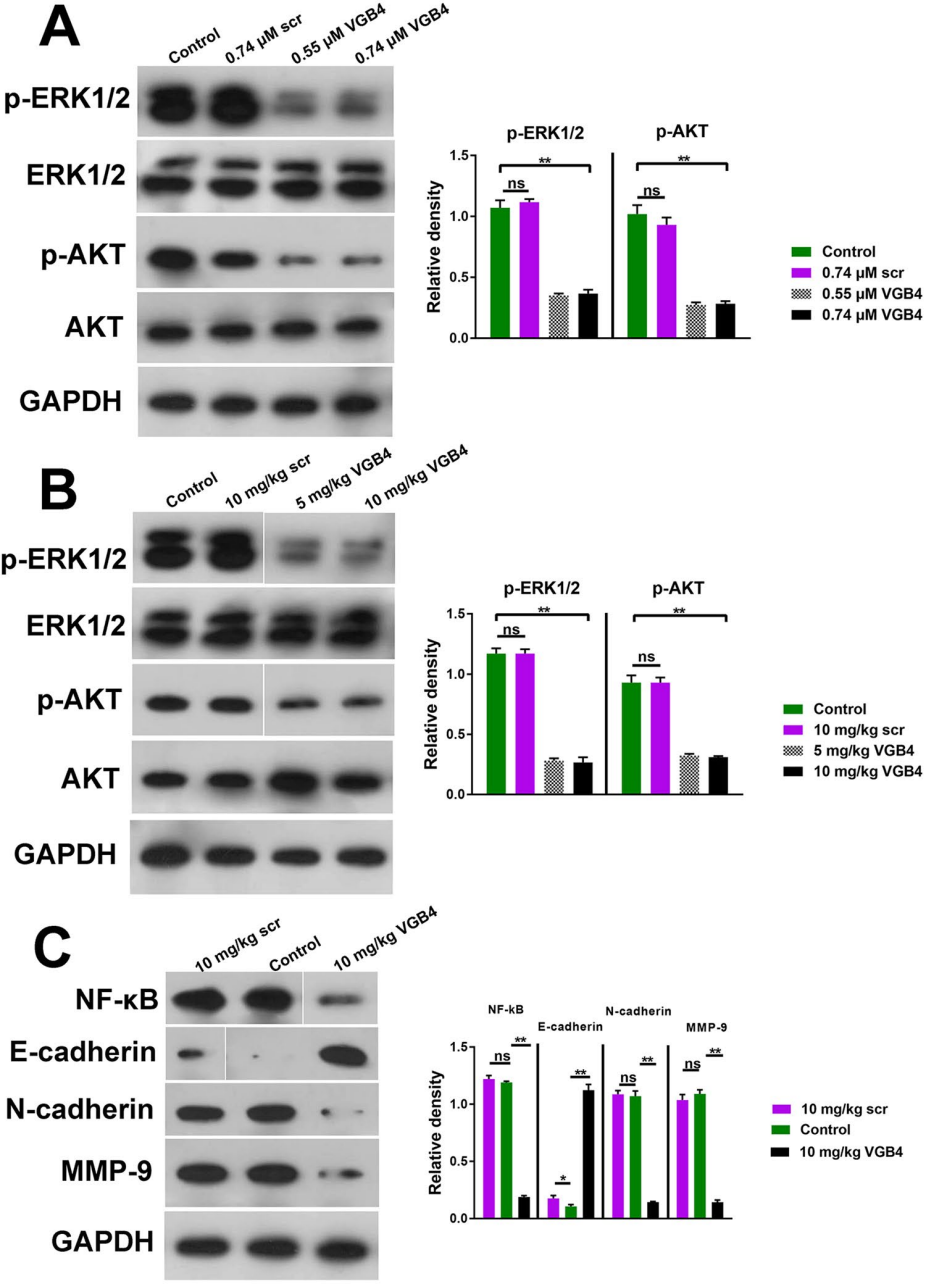
Effect of VGB4 on intracellular signaling pathways. (**A**) HUVECs were treated with VGB4 (0.55 and 0.74 μM) in the presence of VEGF-A (200 ng/ml) and then Cell lysates were subjected to western blot to analyze the expression level of p-ERK1/2, ERK1/2, p-AKT and AKT. GAPDH was used as reference to quantify protein bands by densitometry using ImageJ software. Full-length blots are presented in Supplementary Fig. S1. (**B**) The tumor tissues treated with VGB4 on the last day of the peptide administration (day 28) (5 and 10 mg/kg) were subjected to western blot to analyze the expression level of p-ERK1/2, ERK1/2, p-AKT and AKT. (**C**) 4T1 tumor tissue sections treated with VGB4 (10 mg/kg) were subjected to western blot to analyze the expression level of NF-κB, E-cadherin, N-cadherin and MMP-9. GAPDH was used as reference to quantify the protein bands by densitometry using ImageJ software. (**B**) and (**C**) Full-length blots are presented in Supplementary Fig. S2. All data were expressed as mean ± SD, n = 3, *P ≤ 0.05, **P ≤ 0.001 compared to the control.

Binding of VEGF to VEGFR-1 and VEGFR-2 induces cell proliferation, migration, invasion and angiogenesis through the PI3K/AKT and MAPK/ERK1/2 signaling pathways^29^. To assess the suppressing effect of VGB4 on PI3K/AKT and MAPK/ERK1/2 signaling pathways, western blot analysis of AKT, p-AKT, ERK1/2 and p-ERK1/2 were performed both *in vitro* (HUVECs) and *in vivo* (4T1 tumor tissue sections). The effect of VGB4 and scr peptide on VEGF-induced downstream signaling pathways was examined in HUVECs and the levels of p-ERK1/2 and p-AKT kinases were determined by incubation of HUVECs in the presence of VGB4 (0.55 μM and 0.74 μM) or scr (0.74 μM) in the presence of VEGF-A (200 ng/ml). As shown in Fig. 7A, VGB4 potently blocked both p-ERK1/2 and p-AKT formation compared to control and scr peptide (*P ≤ *0.001). Similarly, VGB4 significantly decreased p-ERK1/2 and p-AKT levels in 4T1 murine MCTs compared to control and scr peptide-treated group (*P ≤ *0.001) (Fig. 7B). These results reveal that downstream signaling of VEGFR1 and VEGFR2 are potently inhibited by VGB4.

Upregulated nuclear factor kappa-light-chain-enhancer of activated B cell (NF-κB) induces loss of E-cadherin expression and matrix metalloproteinase-9 (MMP-9) production in metastatic cells^30^. In addition, the enhanced N-cadherin expression through E-cadherin to N-cadherin shift, known as an Epithelial Mesenchymal Transition (EMT) marker, is associated with the progression and metastasis of tumor cells^31^. To investigate whether VGB4 is also able to block the metastasis of 4T1 cells, western blot analysis of NF-κB, E-cadherin, N-cadherin and MMP-9 expression was performed in tumor tissue sections on the last day of the peptide administration (day 28) (10 mg/kg/day). Our results demonstrated that the expression of NF-κB, N-cadherin and MMP-9 significantly reduced (*P ≤ *0.001) and E-cadherin expression strongly increased (*P ≤ *0.001) compared to control and scr peptide treated groups (Fig. 7C), implying that metastasis-related factors are inhibited by VGB4. Taken together, VGB4 exhibited VEGF antagonistic properties, impeding the VEGF-induced signaling pathways.

## Discussion

Vigorous strategies have been emerged in modulating angiogenesis triggered by VEGF-A and -B, acting through two transmembrane tyrosine kinase receptors VEGFR1 and VEGFR2. Whereas VEGFR2 has been noted as the major positive regulator of angiogenesis^31^, the role of VEGFR1 is controversial. As a negative regulator of angiogenesis, VEGFR1 binds with a high affinity to VEGF-A, resulting in downregulation of VEGFR2 signaling^32–34^. On the contrary, numerous investigations revealed that VEGFR1 is critical for tumor growth and metastasis^35–38^, supporting the notion that the simultaneous blockade of VEGFR1 and VEGFR2 is required for effective suppression of tumor angiogenesis, growth and metastasis^20^. Therefore, barricade of VEGFR1 or VEGFR2 alone seems to be inadequate for VEGF/VEGFR therapy. In the present study, we designed a peptide (referred to as VGB4) that recognized VEGFR1 as well as VEGFR2 and abrogated their downstream signaling, accompanied by a significant inhibition of VEGF-A-induced proliferation, migration and angiogenesis in endothelial cells. In addition, VGB4 accumulated in the tumor tissue and potently inhibited tumor growth and metastasis in a 4T1 mammary carcinoma model.

For simultaneous blockade of VEGFR1 and VEGFR2, the VEGFR1D2 binding sites of VEGFB as well as VEGFR2D2 and -D3 binding sites of VEGFA were linked into a single molecular entity. Cell-based binding studies revealed that VGB4 recognizes and blocks VEGFR1 and VEGFR2 on the surface of HUVECs. The inhibition of proliferation of HUVEC, 4T1 mammary carcinoma cell line that both mostly express VEGFR1 and scarcely VEGFR2, and U87 glioblastoma cell line that highly express VEGFR2 and no VEGFR1^39–41^, further confirmed the dual specificity of VGB4 for VEGFR-1 and -2.

The blockage of VEGF/VEGFRs interaction led to the inhibition of angiogenesis and tumor growth. VGB4 markedly prevented VEGF-driven angiogenesis by targeting major aspects of endothelial cell functions, i.e. proliferation, migration and tube formation. Based on a recent report by Wang *et al*.^19^, a peptide mimicking loop1 of VEGFB (named Peptide 18) inhibited EC tube formation with IC_50_ value of 10 μM when stimulated with 30 ng/ml of VEGF. By comparison, VGB4 inhibited two-dimensional tube formation at approximately twenty-fold lower IC_50_ value of ~0.5 μM even in the presence of a very high concentration of VEGF (200 ng/ml). The marked advanced antiangiogenic property of VGB4 compared to Peptide 18 could be due to the fact that Peptide 18 comprising VEGF-B residues led to the suppression of VEGFR1 downstream signaling, whereas VGB4 composed of VEGFR1-binding residues from VEGF-B and VEGFR2-binding residues from VEGF-A resulted in the blockade of both VEGFR1- as well as VEGFR2-mediated signaling. VEGFR1 and VEGFR2 primarily activate PI3K/AKT and MAPK/ERK signaling pathways, respectively^29^. Accordingly, we evaluated the inhibitory effect of VGB4 on the signaling through PI3K/AKT and MAPK/ERK1/2 pathways by western blot analysis of the levels of p-AKT and p-ERK1/2 both *in vitro* and *in vivo* conditions. In HUVECs, VGB4 significantly suppressed the activation of AKT and ERK1/2, which confirms the blockade of both VEGFR1 and VEGFR2-mediated signaling pathways. Our *in vivo* studies demonstrated that VGB4 accumulated in murine 4T1 MCTs and led to a dose-dependent regression of 4T1 MCT growth with the maximal effect at a dosage of 5 mg/kg. In line with this, *in vivo* western blot analyses showed a marked decrease of p-ERK1/2 and p-AKT levels in VGB4-treated compared to PBS-treated tumors, which further supports the VEGF antagonizing property of VGB4. Moreover, the significant inhibition of NF-κB, MMP-9 and N-cadherin as well as upregulation of E-cadherin in the treated tumors revealed anti-invasion and anti-metastatic activity of VGB4. VEGFR1 and -R2 activation mediates VEGFR phosphorylation^42^. Our immunohistochemical analyses revealed that administration of VGB4 markedly inhibited the phosphorylation of both VEGFR1 and -R2 in a dose-dependent manner. Since angiogenesis is a general sign of cancer and plays a key role in breast cancer metastatic properties, we further investigated the effect of VGB4 on this hallmark. Immunohistochemical studies showed a dose-dependent reduction of MVD (CD31 and CD34 expression) in VGB4-treated tumors, which is consistent with its *in vitro* antiangiogenic properties. These *in vitro* and *in vivo* results indicate that VGB4-driven inhibition of tumor growth is due to its anti-angiogenic effects. The loss of blood supply led to the decrease in the number of proliferating tumor cells. In particular, by taking into consideration that ERK1/2 mediates cell proliferation^43^, the reduced Ki-67 expression in VGB4-treated tumors is attributable to the inhibitory effect of VGB4 on ERK1/2 phosphorylation. As mentioned earlier, VGB4 significantly inhibited the proliferation of 4T1 cells *in vitro* and the expression level of Ki-67 (index of cell proliferation) *in vivo*, suggesting that the antagonistic effect of VGB4 might be also due to its anti-tumor cell effects. On the other hand, a dose-dependent downregulation of Bcl2 along with the upregulation of P53 and increased TUNEL staining revealed that antiangiogenic effects of VGB4 is also executed by the induction of apoptosis in tumor tissues. The induction of apoptosis by VGB4 can be associated with the suppression of p-AKT, which is shown to promote apoptosis^44^.

## Conclusion

Our peptide designing strategy based on the receptor-binding segments of VEGF-B and VEGF-A led to the abrogation of the angiogenesis signaling factors, i.e. VEGFR1 and -R2, AKT and ERK1/2, followed by a notable decrease in tumor cell proliferation (Ki67 expression) and angiogenesis (expression of CD31 and CD34), and also caused promotion of apoptosis (increased TUNEL staining and P53 expression and decreased Bcl-2 expression) and a significant decrease in metastasis-related factors (increased E-cadherin and decreased N-cadherin, NF-κB and MMP-9 expression). According to these results, VGB4 could be considered as a therapeutic target for cancer treatment.

## Materials and Methods

### Synthetic peptide and reagents

The peptide was synthesized and purified by high-performance liquid chromatography to a purity of 85%, analyzed by matrix-assisted laser desorption/ionization time-of-flight mass spectrometry (MALDI-TOF), and confirmed by electrospray ionization mass spectrometry (ESI-MS) analysis (Shine Gene Biotechnologies, Inc., Shanghai, China). Anti-AKT (Ab25893), anti-AKT phospho S473 (Ab81283), anti-VEGFR2 (Ab9530), FITC-secondary anti-mouse (Ab6724), anti-VEGFR1 (Ab11934), PE-secondary anti-mouse (Ab97024), anti-phospho-VEGFR2 (Ab194806), anti-CD31 (Ab32457), anti-CD34 (Ab81289), anti-Ki-67 (Ab15580), anti-p53 (Ab131442), and anti-Bcl2 (Ab59348) were purchased from Abcam, Cambridge, UK; anti-P44/p42 MAPK (ERK1/2) (9102S) and anti-phospho-p44/p42 MAPK (ERK1/2) (Thr202/Tyr204) (4377S) were purchased from Cell Signaling Technology, Danvers, Massachusetts, USA; anti-MMP-9 (SC-6840), anti-N-cadherin (SC-7939) and anti-NF-κB (SC-8008) were purchased from Santa Cruz Biotechnology INC, California, USA; anti-E-cadherin (PM170AA) was purchased from Biocare Medical, USA; TUNEL assays were performed using an *in situ* Cell Death Detection Kit POD (Roche Diagnostic GmbH, Germany). Geltrex^TM^ LDEV-Free Reduced Growth Factor Basement Membrane Matrix (A14132–02) was purchased from Gibco Grand Island, NY, USA; Rat tail collagen type I (2 mg/ml in 0.5 M acetic acid), DAPI (D9542), Propidium iodide (P4170), RIPA buffer, and polyvinyl difluoride (PVDF) membranes (IPVH00010) and anti-phospho-VEGFR1 (SAB4504006) were from Sigma (St Louis, MO, USA). Dextran-coated cytodex 3-microcarriers and ECL (RPN2109) were from Amersham Pharmacia Biotech (Piscataway, NJ, USA).

### Cell culture

Human Umbilical Vein Endothelial Cell (HUVEC; NCBI, C554), 4T1 mammary carcinoma cell line (NCBI, C604) and Human glioblastoma U87 MG cell line (NCBI, C531) were purchased from the National Cell Bank, Pasteur Institute of Iran. HUVECs and U87 cells were cultured in Dulbecco’s Modified Eagle’s Medium (DMEM; Gibco, Life Technologies, USA); 4T1 cells were cultured in RPMI-1640 medium (Gibco Grand Island, NY, USA). All media were supplemented with 10% fetal bovine serum (FBS; Sigma, St. Louis, Missouri, USA), 100 IU/mL penicillin G and 100 μg/mL streptomycin. The cultures were incubated at 37 °C and in a humidified atmosphere of 5% Co_2_ until 90% confluent. All subsequent experiments were done with low passage number (passages 3–5).

### Construction of the structural model

The structure of residues 1–4 and residues 10–23 extracted from the entire VEGF-B structure (PDB ID: 2XAC), and the structure of residues 5–9 extracted the entire VEGF-A structure (PDB ID: 3V2A) were covalently linked into a single molecular entity referred to as VGB4. the structural model for VGB4 was constructed using the MODELLER program Ver. 9v2^45^.

### Conjugation of peptides to FITC

To prepare the fluorescent probe FITC-peptide, FITC was coupled to the amine group of the N-terminus. FITC was dissolved in DMSO, getting 1 mg/ml solution, which was added into 1 mg/ml the peptides. The reactions were conducted in pH 8.5 to reduce the reaction with side groups of lysine and arginine. The tube wrapped in foil and incubated in 37 °C for 90 min. To remove unreacted FITC and peptides and exchange the peptide into the storage buffer (PBS), the reaction mixture was loaded onto an equilibrated Sephadex G10 column (1.5 × 150 cm) and eluted with PBS buffer, pH 7.5. The concentrations of the collected samples were determined based on the absorbance at 280 nm and using the following equation (http:// www.sigmaaldrich.com):

Peptide (mg/ml) = (A280 x DF x MW)/e

DF (dilution factor), MW (the peptide molecular weight) and e (the molar extinction coefficient of each chromophore at 280 nm).

### Binding assays

To assess the ability of VGB4 in binding to endothelial cells, 3 × 10^4^ HUVECs/well were incubated with FITC-labeled VGB4 peptide (0.37, 0.55 and 0.74 μM) or FITC-labeled scr peptide (0.74 μM) in DMEM medium containing 2% FBS for overnight in the dark. After three times washing with PBS, cells were trypsinized and resuspended in PBS for flow cytometric analysis with a BD FACSCalibur Flow Cytometer.

To assay the binding of VGB4 to VEGFR1, 5 × 10^4^ HUVECs were seeded in 12-well plate and let to grow (~80% confluent), then incubated with the different concentrations of VGB4 (0.37, 0.55 and 0.74 μM) and scr peptide (0.74 μM) for overnight. After washing each well two times with PBS-Tween (0.05% Tween), the cells were fixed with 4% formaldehyde for 20 minutes at room temperature (RT). After washing two times with PBS-T, blocking buffer containing 1% BSA/ 10% normal goat serum/0.3 M glycine in 0.1% PBS-T was added for 1 h. In the next step, the cells were incubated overnight at 4 °C with anti-VEGF Receptor 1 primary antibody. After four times washing, Phycoerythrin (PE)-labeled goat anti-mouse IgG secondary antibody was added. Then, the cells were counterstained with DAPI and were observed under a fluorescence microscope. For further investigation of VGB4 binding to VEGFR1, flow cytometric analysis was also performed using a BD FACSCalibur Flow Cytometer.

Examination of the binding of VGB4 to VEGFR2 was performed the same as described above. HUVECs were incubated overnight at 4 °C with anti-VEGFR2 antibody. After four times washing, Fluorescein isothiocyanate (FITC)-labeled rabbit anti-mouse IgG secondary antibody was added and then incubated in the dark situation at 37 °C for 1 h. After washing, the cells were counterstained with the nuclei staining dye propidium iodide (PI) and were observed under a fluorescence microscope. For further investigation of VGB4 binding to VEGFR2, flow cytometric analysis was also performed using a BD FACSCalibur Flow Cytometer. All images were analyzed using Image J software (NIH Image, National Institutes of Health; online at: http://rsbweb.nih.gov/ij/).

### Cell proliferation assay

2–3 × 10^3^ HUVEC, 4T1 or U87 glioblastoma cell-lines were cultured in DMEM medium supplemented with 10% FBS at 37 °C with 5% CO_2_ in 96-well plate. After 24 h, The cells were incubated in the media supplemented with 2% FBS, different concentrations (0.09, 0.18, 0.37, 0.55 and 0.74 μM) of VGB4 or (0.74 μM) scr in the presence of (200 ng/ml) VEGF-A. After 36 h incubation, 3-(4,5–dimethylthiazol-2-yl)-2,5-diphenyl tetrazolium bromide (MTT) was added to each well and the plates were incubated in the dark situation at 37 °C for 4 h. Then the insoluble purple formazan product was dissolved by dimethyl sulfoxide (DMSO). Absorbance was measured at 570 nm using an ELISA plate reader. The assay was performed in triplicate.

### Wound healing assay

3 × 10^3^ HUVECs were grown to confluence in 96-well plate. The monolayer was mechanically wounded using a sterile pipette tip followed by washing with PBS. The cells were incubated in the serum-starved DMEM medium containing 2% FBS and different concentrations (0.37, 0.55 and 0.74 μM) of VGB4 or (0.74 μM) scr in the presence of (200 ng/ml) VEGF-A for 24 h. Then HUVECs were washed with PBS for two times and fixed using 4% paraformaldehyde at RT. After staining the cells with Giemsa, the cells were photographed by the camera connected to an inverted microscope. The number of migrated cells were microscopically assessed. The assay was performed in triplicate.

### Angiogenesis assays

Two tube formation methods were used. One was based on tube-like structure formation on Geltrex. Geltrex^TM^ LDEV-Free Reduced Growth Factor Basement Membrane Matrix was thawed on ice at 4 °C. Then (50 μl) Geltrex^TM^ was added to each well of a 96-well plate and incubated at 37 °C for 30 min to allow to solidify. 14 × 10^3^ HUVECs were seeded on the layer of Geltrex^TM^ and incubated for 5 h at 37 °C. The medium was replaced with 200 μl of starved DMEM medium supplemented with 2% FBS and different concentrations (0.37, 0.55 and 0.74 μM) of VGB4 or (0.74 μM) scr in the presence of (200 ng/ml) VEGF-A. The plate was incubated at 37 °C with 5% CO_2_. After 14 h incubation, tube formation quality of control and treatment was evaluated under invert microscope. Total tube length and tube number were quantified by ImageJ software. The other was the micro carrier bead sprouting assay. HUVECs were mixed with sterilized cytodex-3 micro carrier beads and incubated for overnight at 37 °C. EC-covered beads were embedded into the collagen gel and then DMEM supplemented with 2% FBS with a range of concentrations (0.37, 0.55 and 0.74 μM) of VGB4 or (0.74 μM) scr in the presence of (200 ng/ml) VEGF-A were added on top of the collagen gel. After 24 h, angiogenesis was monitored microscopically. All the capillary-like structures were photographed by a digital camera and the number of sprouts out growth on beads were quantified by ImageJ software. The assays were performed in triplicate.

### *In vivo* antitumor efficacy evaluation

The animal study was performed according to Institutional Animal Care and Use Committee (IACUC) of Tehran University of Medical Sciences. All protocols were approved by the IACUC of Tehran University of Medical Sciences. Murine 4T1 MCTs were excised from BALB/c mice-bearing breast cancer and cut into fragments (approximately 5 mg) and then were subcutaneously transplanted in to the right flank of 4–6 week-old female BALB/c mice under ketamine (100 mg/kg, i.p.) and xylazine (10 mg/kg, i.p.) anesthesia. When the tumor volume reached ~ 200 mm^3^, tumor 4t1 BALB/c mice were randomly divided to two groups (n = 7 mice/group). Daily intraperitoneal injection of VGB4 at different doses (0.25, 1, 2.5, 5 and 10 mg/kg) or scr (10 mg/kg) was administrated for treated groups whereas the control group received the equal volume of PBS for two weeks. To monitor the growth of the tumor, length and width of the tumors were measured every other day with digital caliper and the tumor volume was calculated by using the formula^46^. Volume = 0.52 × length × width^2^.

### *In vivo* imaging

To confirm that VGB4 peptide accumulated in 4T1 tumor, *in vivo* fluorescence imaging using fluo vision optical imaging system was performed after preparation of BALB/c mice-bearing breast cancer. In this non-invasive imaging technique, mice received the same anesthetic dose (described above). After intravenous VGB4-FITC or scr-FITC injection into the BALB/c mice, the whole body of each mouse was scanned. Fluorescence images were taken at 0, 30, 60 and 90 minutes after injection. Fluorescence intensity of FITC in tumor region was analyzed using Amide software (Source Forge; http://amide.sourceforge.net).

### Immunohistochemistry and TUNEL staining

Five-micrometer-thick formalin-fixed, paraffin embedded tissue sections of treated and untreated mice were de-paraffinized with xylene and rehydrated through graded ethanol. Tumor tissue sections were stained with Hematoxylin and eosin (H&E) for investigation of morphological changes in treatment and control groups. IHC staining analysis was performed to localize specific tissue antigens. The sections were incubated at 4 °C with the primary mouse monoclonal antibodies for CD31, CD34, Ki-67, Bcl2, p53, Phospho-VEGFR1 and Phospho-VEGFR2 for overnight. Antigens were detected with 3,3-diaminobenzidine (DAB). Furthermore, to evaluate apoptosis induction, the terminal deoxynucleotidyl transferase (TdT) dUTP Nick-End Labeling (TUNEL) assay was performed using the *in situ* cell death detection kit. All images were analyzed using Image J software.

### Western blot

4T1 tumor tissues or HUVECs from treated and untreated groups were lysed with RIPA buffer containing protease and phosphatase inhibitor. After centrifugation, protein concentration was determined by Lowry method. Equal amounts of total proteins were subjected to 12% sodium dodecyl sulfate-polyacrylamide gel electrophoresis. Proteins on gel were transferred to polyvinyl difluoride (PVDF) membranes followed by blocking with 5% non-fat milk for 2 h. The primary antibodies including anti-phospho-ERK1/2, anti-total-ERK1/2, anti-phospho-AKT, anti-total-AKT, anti-NF-κB, anti-E-cadherin, anti-N-cadherin, anti-MMP-9 and anti-GAPDH were incubated overnight at 4 °C. After three times washing in Tris Buffered Saline-Tween (TBS-T), the membranes were incubated with an appropriate HRP-conjugated secondary antibody for 1 h at room temperature. Protein bands were visualized using ECL reagent. The densitometry was normalized to GAPDH.

### Statistical analyses

Statistical analyses were performed using SPSS 19.0 software. After assessing data normality with Kolmogorov-smirnov test, Unpaired Student’s t-Test and One-Way ANOVA with Duncan’s post hoc test were used to evaluate the significant differences respectively between two and more than two groups. *P ≤ *0.05 was considered as significant differences.

## Electronic supplementary material

supplementary information

## Data Availability

All data generated or analysed during this study are included in this published article (and its Supplementary Information file).
