# Supplementary material for: A peptide mimicking the binding sites of VEGF-A and VEGF-B inhibits VEGFR-1/-2 driven angiogenesis, tumor growth and metastasis
Source: Sci Rep. 2018 Dec 18;8:17924. doi: 10.1038/s41598-018-36394-0 (PMC6298961; doi:10.1038/s41598-018-36394-0)
Supplement: Supplementary file 1 — supplementary information [file 41598_2018_36394_MOESM1_ESM.pdf]

Supplementary information

A peptide mimicking the binding sites of VEGF-A and VEGF-B inhibits VEGFR-1/-2 driven angiogenesis, tumor growth and metastasis

Maryam Farzaneh Behelgardi, Saber Zahri, Farhad Mashayekhi, Kamran Mansouri, S. Mohsen Asghari

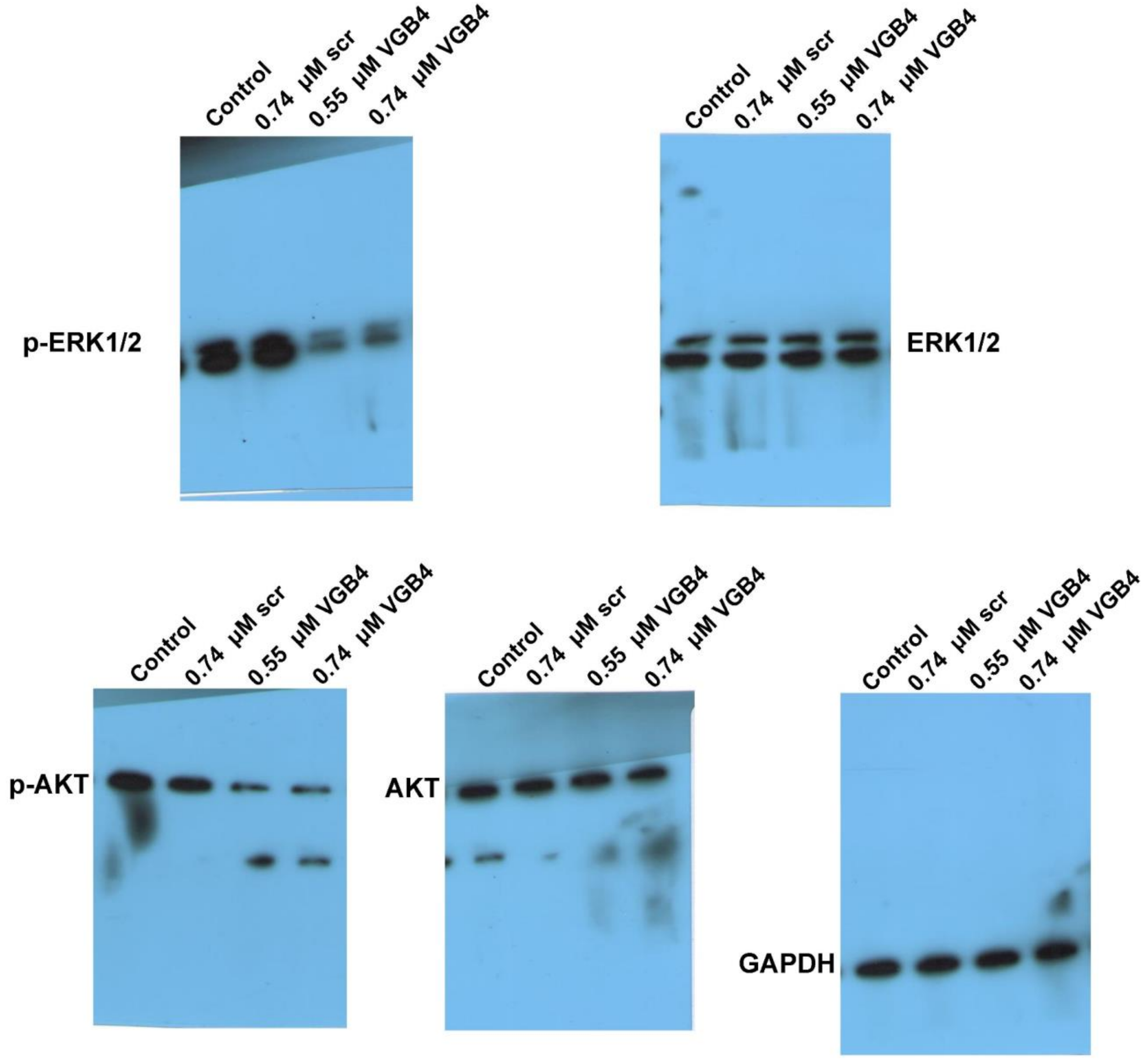

Figure S1: Full length blots of p-ERK1/2, ERK1/2, p-AKT, AKT and GAPDH in HUVECs with or without VGB4 (shown as cropped images in Figure 7A).

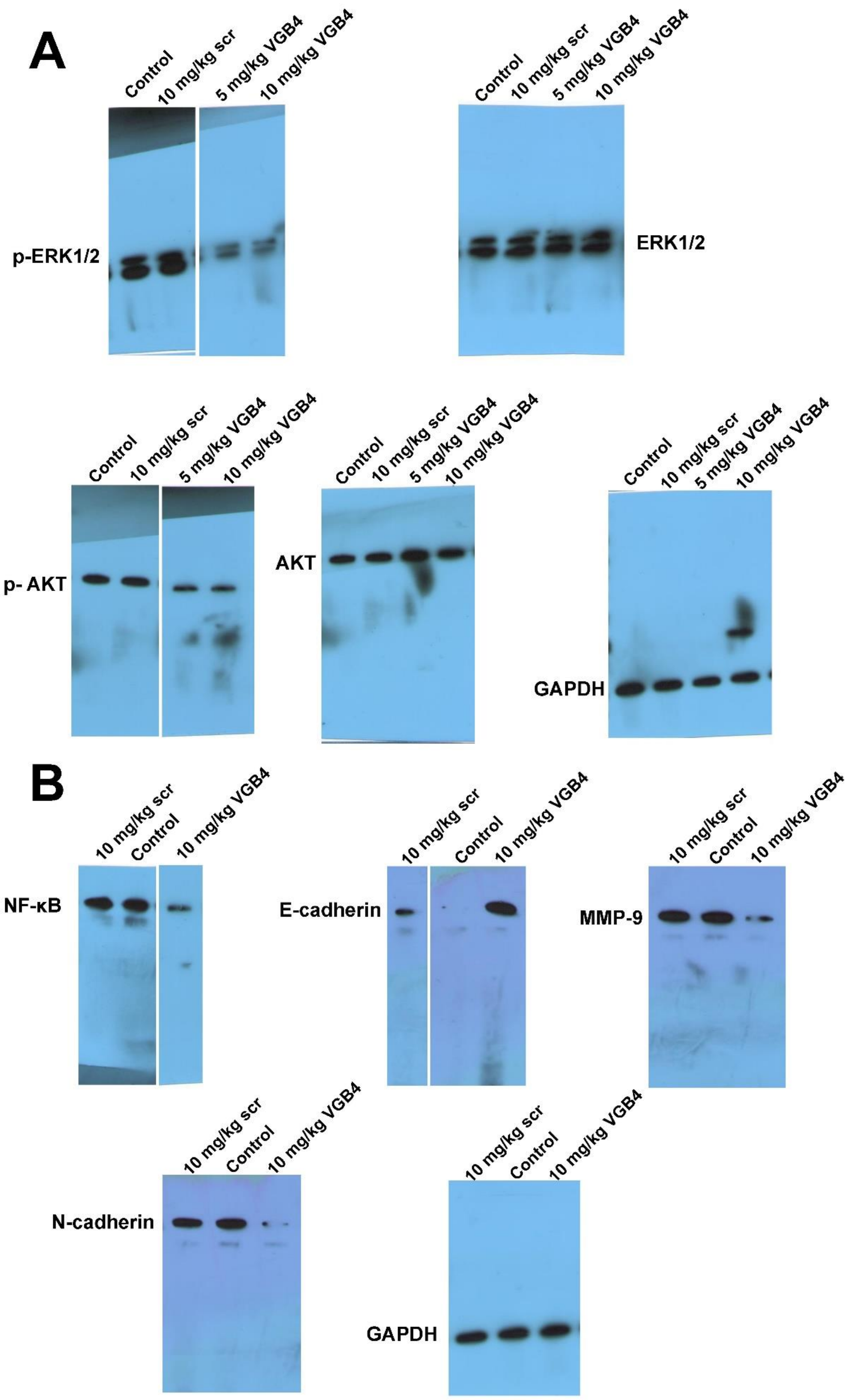

Figure S2: (A) Full length blots of p-ERK1/2, ERK1/2, p-AKT, AKT and GAPDH, and (B) Full length blots of NF-κB, E-cadherin, N-cadherin, MMP-9 and GAPDH in VGB4 treated tumor tissue sections and control group (shown as cropped images in Figure 7B and C).
